# Screening Depression, Falls, and Fear of Falling in Older Adults: Create Community Partnerships

**DOI:** 10.1093/geroni/igaf122.2640

**Published:** 2025-12-31

**Authors:** Veronica Decker, Jethro Raphael Suarez, Yi Liu, Janet Lopez, Michael Dino, Dahee Kim, Chitra Banarjee, Ladda Thiamwong

**Affiliations:** University of Central Florida, Orlando, Florida, United States; University of Central Florida, Florida, United States; University of Central Florida, Orlando, Florida, United States; University of Central Florida, Orlando, Florida, United States; University of Central Florida, University of Central Florida, Florida, United States; University of Central Florida, Orlando, Florida, United States; University of Central Florida, Orlando, Florida, United States; University of Central Florida, Orlando, Florida, United States

## Abstract

The biopsychosocial impacts of falls have been considered a public health crisis. Fear of falling (FoF) is rarely screened in practice despite its impact on healthy aging. This may cause social isolation and depression, increasing FoF and fall incidents and leading to restrictions on engaging in physical and social activities among older adults. There has been a need to investigate a multitude of factors contributing to falls, the unique stressors influencing FoF, and the progression and interplay of depression in low-income older adults. This study aimed to (1) screen for depression, falls, and FoF among older adults recruited through community center partnerships and (2) examine the associations between such variables. We screened 301 older adults ($\bar{\text{x}}$ = 74, SD = 7.29 years) for depression with the Patient Health Questionnaire-9, FoF with the Short Falls Efficacy Scale-International, and self-reported fall incident. Binary logistic regression was used to examine whether depression is significantly associated with FoF and the number of falls over the past year. Participants who experienced 2 or more falls were twice as likely to indicate depression relative to those with 0 falls (OR = 2.06). Participants with moderate FoF were over 3 times more likely to report depression (OR = 3.21) and those with a high FoF were over 7 times (OR = 7.60) more likely. Results show that a biopsychosocial approach is needed to address the complex relationship between depression, falls, and FoF. Future research should encourage partnerships with local communities to provide screenings and education to underrepresented older adults, focusing on reducing falls and FoF.

